# Supplementary material for: Non-invasive spinal cord neuromodulation enables volitional anti-gravity leg movements after motor-complete spinal cord injury: responders vs. non-responders
Source: J Neuroeng Rehabil. 2026 May 18;23:216. doi: 10.1186/s12984-026-02011-9 (PMC13371260; doi:10.1186/s12984-026-02011-9)
Supplement: Supplementary file 1 — Supplementary Material 1. [file 12984_2026_2011_MOESM1_ESM.pdf]

Supplemental Online-Only Figures

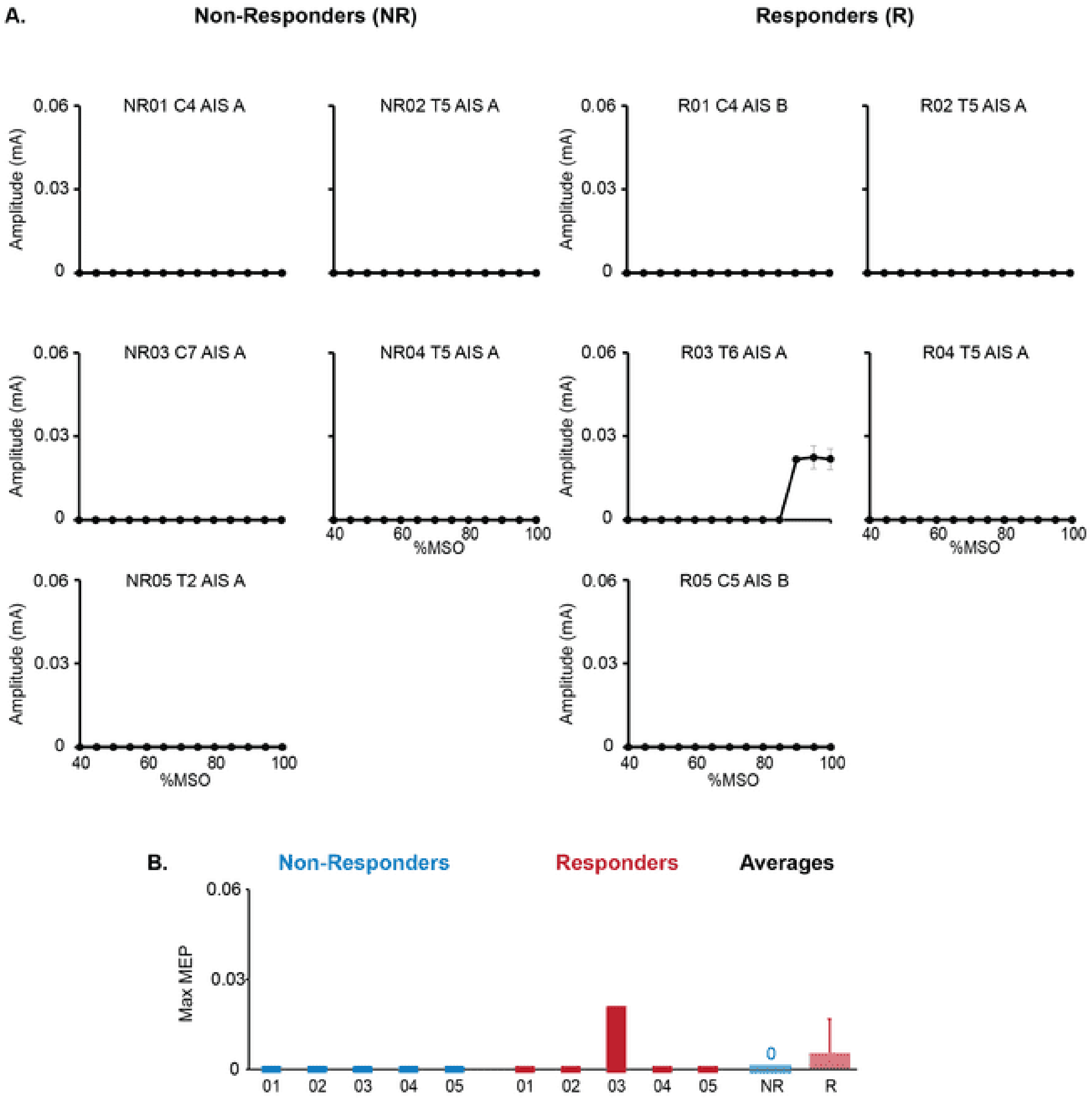

Supplemental Figure 1: Motor evoked potentials
(A) Recruitment curves of motor-evoked potentials in the Tibialis Anterior, elicited by Transcranial Magnetic Stimulation over the motor cortex for each responder and non-responder. **(B)** The maximum MEP amplitude for each participant and group averages with tSCS off.

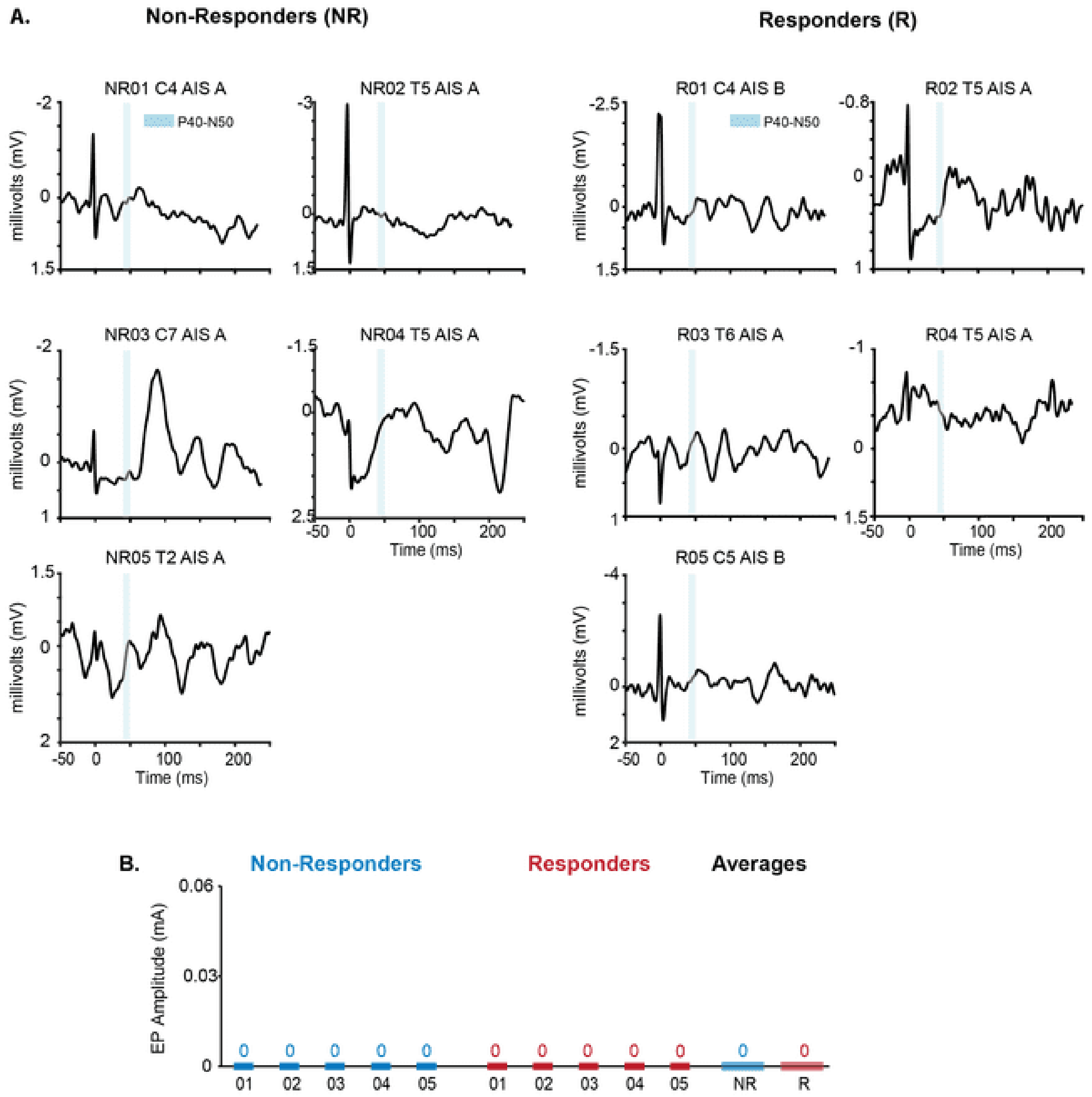

Supplemental Figure 2: Somatosensory evoked potentials

(A) Tibial nerve SEP traces and P40-N50 amplitudes (blue shaded region) recorded from Cz referenced to Fz for each responder (R) and non-responder (NR). **(B)** P40-N50 amplitude for each participant and group averages with no spinal cord stimulation.

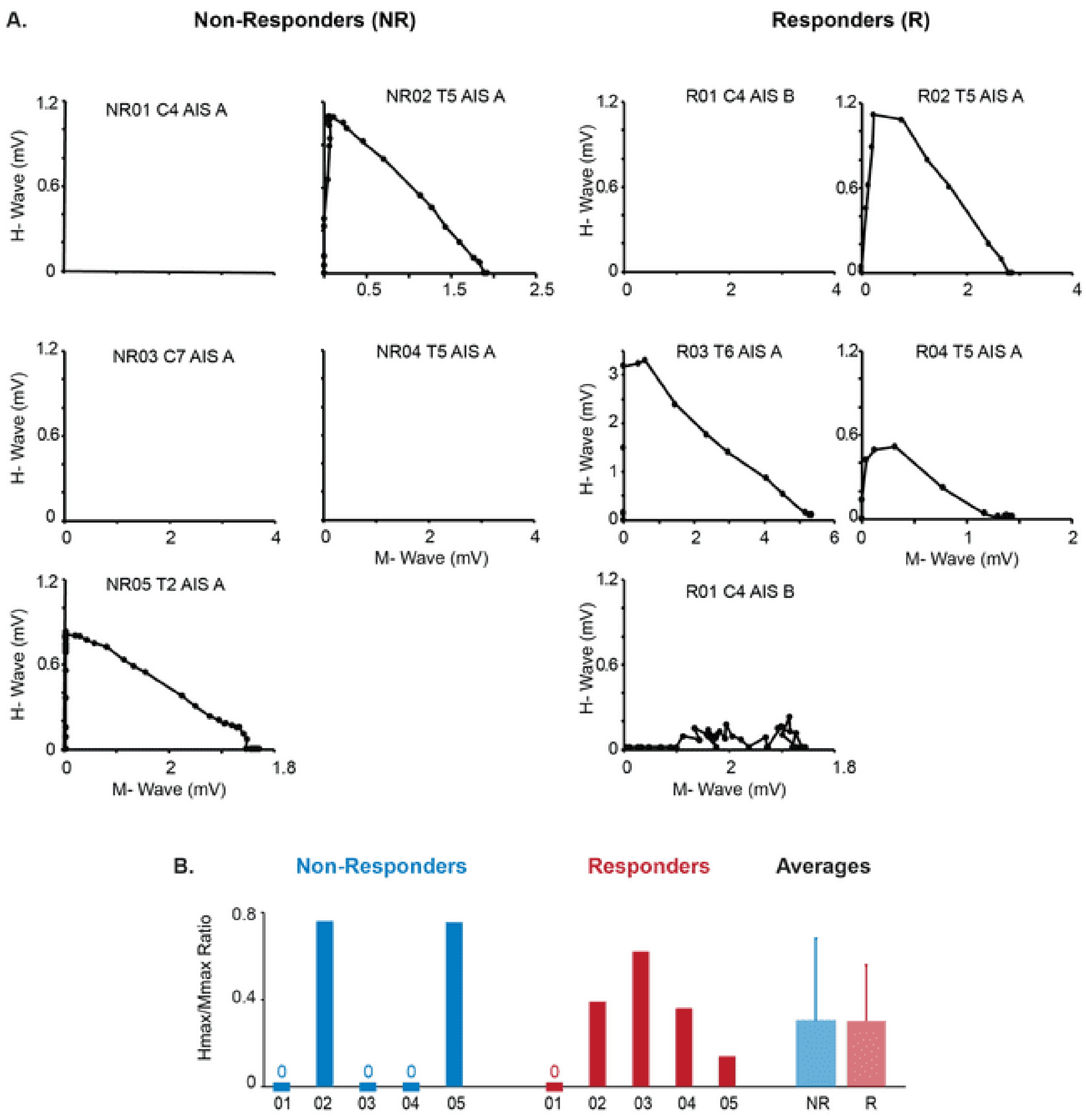

Supplemental Figure 3: Hoffman (H) reflex

(A) Soleus H-reflex versus M-wave for each responder (R) and non-responder (NR). **(B)** Hmax/Mmax ratio for each participant and group averages with with no spinal cord stimulation.

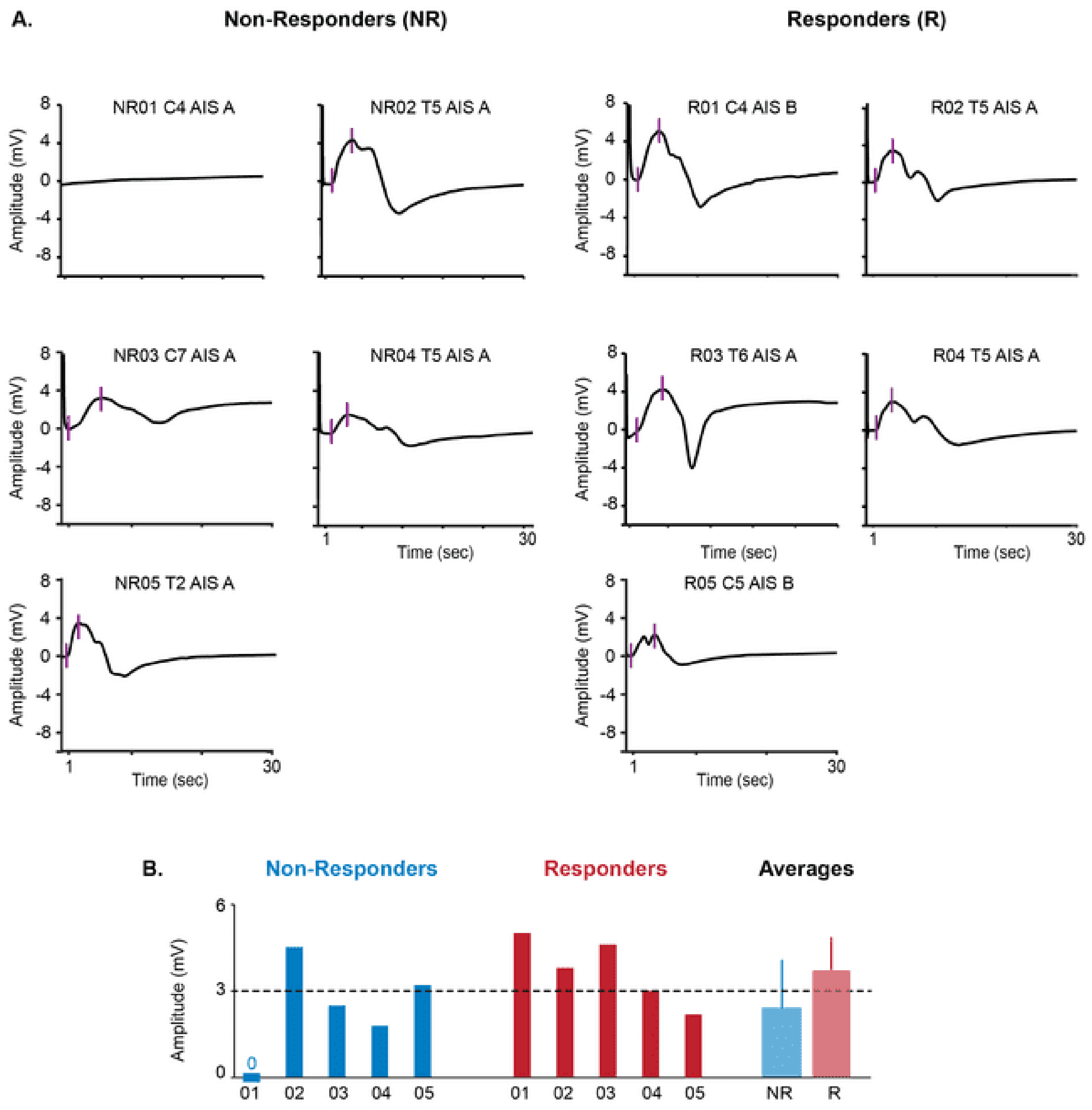

Supplemental Figure 4: Compound muscle action potentials (CMAPs)
(A) Fibular nerve CMAP waveforms for each responder and non-responder. **(B)** Quantified CMAP amplitudes for each participant and group averages with no spinal cord stimulation. Dotted line represents normative amplitudes.

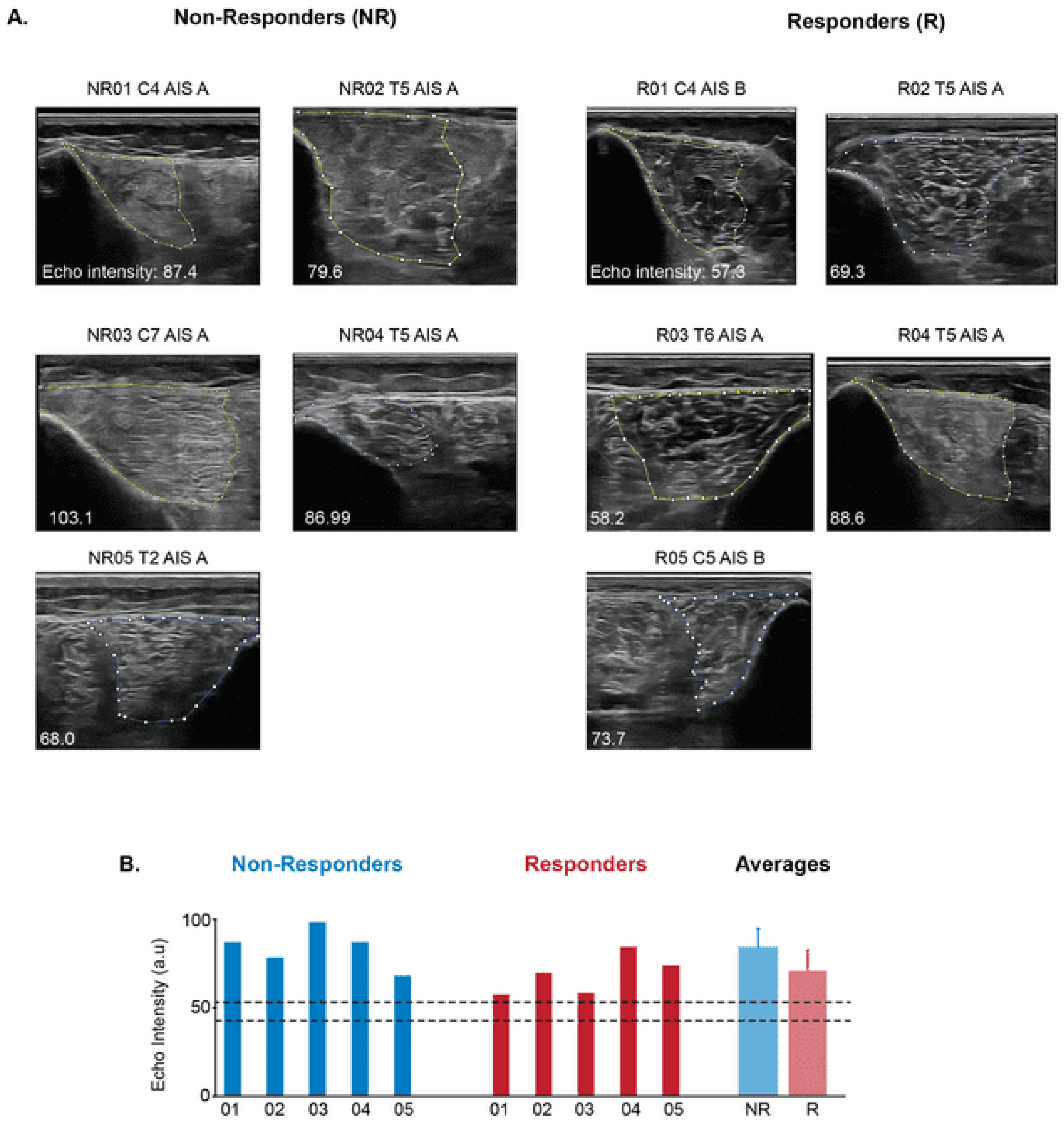

Supplemental Figure 5: Muscle morphology
(A) Ultrasound images of the tibialis anterior for each responder and non-responder. **(B)** Quantified echo-intensity values for each participant and group averages with tSCS off. Dotted lines indicate 95% confidence intervals from healthy controls (see Methods).
